# Development and validation of claims-based algorithms to identify interstitial lung disease among Japanese patients with cancer in routine clinical practice using real-world data sources

**DOI:** 10.3389/fonc.2026.1750236

**Published:** 2026-09-09

**Authors:** Yu-Jing Huang, Toshihiro Takeda, Yoshie Shimai, Taizo Murata, Kento Sugimoto, Yoshito Takeda, Haruhiko Hirata, Makoto Yamamoto, Midori Yoneda, Seigo Minami, Masahiro Higashi, Alan James Michael Brnabic, Zbigniew Kadziola, Tsutomu Kawaguchi, Yucherng Chen, Yasushi Matsumura

**Affiliations:** 1 Global Patient Safety, Pharmacoepidemiology, Eli Lilly and Company, Indianapolis, IN, United States; 2 Department of Medical Informatics, Graduate School of Medicine, Osaka University, Osaka, Japan; 3 Department of Medical Informatics, Osaka University Hospital, Osaka, Japan; 4 Department of Respiratory Medicine and Clinical Immunology, Graduate School of Medicine, Osaka University, Osaka, Japan; 5 Department of Respiratory Medicine, NHO Osaka National Hospital, Osaka, Japan; 6 Department of Radiology, NHO Osaka National Hospital, Osaka, Japan; 7 Global Statistical Sciences, Eli Lilly and Company, Indianapolis, IN, United States; 8 Japan Drug Development and Medical Affairs, Eli Lilly Japan K.K., Kobe, Japan; 9 NHO Osaka National Hospital, Osaka, Japan

**Keywords:** health administrative data, lung diseases, interstitial, machine learning, natural language processing, outcome validation, real-world data

## Abstract

**Introduction:**

Efforts to validate claims-based algorithms for identifying patients with interstitial lung disease (ILD), an important safety concern in Japan, are limited.

**Purpose:**

We developed and validated a claims-based algorithm for identifying ILD in Japanese patients with cancer using machine-learning modeling.

**Methods:**

This Japanese observational study used administrative claims and electronic medical record data collected in January 2013–March 2019 (Phase 1) and January 2015–March 2021 (Phase 2). Patients were classified as ILD cases based on chest computed tomography reports using natural language processing, with confirmatory reviews (ILD_CT+_). Machine-learning modeling strategies (logistic regression, least absolute shrinkage and selection operator [LASSO] logistic regression, and eXtreme Gradient Boosting) selected ILD identification variables from prespecified candidates. Model performances were estimated. Approximately 30% of randomly selected ILD_CT+_ cases were adjudicated using medical records; algorithm performance was adjusted using adjudication results. The best-performing algorithm was validated using an external claims database.

**Results:**

Among 13,601 eligible patients, 415 were ILD_CT+_ cases; 123 were selected for adjudication. The best-performing model was the LASSO reduced model (using only the top variables identified in the full model) (sensitivity: 33.5%; specificity: 99.3%; positive predictive value [PPV]: 76.7%); identified variables were confirmed ILD diagnosis codes, Krebs von den Lungen-6/serum surfactant protein-D codes, age, and sex. This model showed similar performance in an external database (sensitivity: 19.8%; specificity: 99.4%; PPV: 65.5%), when a cutoff of 0.5 was used as a threshold to classify patients per their modeled probability of having ILD.

**Conclusions:**

Despite limited sensitivity, this validated algorithm’s acceptable PPV may enable confident identification of true positive cases of ILD when suspected positive from claims data in Japanese patients with cancer, potentially making it valuable as a case-confirmation tool for retrospective studies using healthcare claims databases, particularly for those comparing relative risks between treatments.

## 1 Introduction

Interstitial lung disease (ILD) describes a group of diseases characterized by lung inflammation and/or fibrosis (1). Higher incidence and prevalence of ILD were reported in Japan versus other countries, possibly due to differences in genetic susceptibility, medical care, adverse event reporting, and regulatory practices (2–4). As a result, ILD is an important safety outcome in Japan, and ILD risks between cancer treatments are of particular interest.

The use of healthcare databases in post-marketing surveillance (PMS) has been endorsed by the Japanese regulatory authority since 2017 (5). While healthcare databases are valuable sources of longitudinal real-world data for pharmacoepidemiology (6), they can be incomplete and do not always capture patients’ medical status accurately. Therefore, validation of outcome definitions and timely publication of the methodology and results are recommended to ensure transparency and promote appropriate evaluation of the results and applications of PMS findings. To date, claims-based algorithms for ILD have been validated using prespecified, rule-based approaches in two studies conducted in the United States (7, 8). However, as diagnostic coding systems and management strategies can vary across countries and regions, and as both studies focused on patients with rheumatoid arthritis, these validated algorithms may not be directly applicable to Japan or to patients with cancer. Therefore, validation of a claims-based algorithm for ILD for use in future PMS studies in Japan is essential.

Traditional approaches for outcome validation often rely on fixed-rule algorithms and goodness-of-fit tests, offering interpretability (9, 10). Although not inherently immune to bias or overfitting (11, 12), machine-learning modeling can offer advantages for handling large numbers of candidate predictors, and for making an objective, data-driven variable selection (9, 13, 14). Using machine-learning modeling, we developed and validated claims-based algorithms for ILD in Japanese patients with cancer to provide a case-confirmation tool for ILD that can be used in retrospective studies using administrative claims data to assess relative ILD risk between treatments.

## 2 Materials and methods

### 2.1 Study design

**Figure 1:**
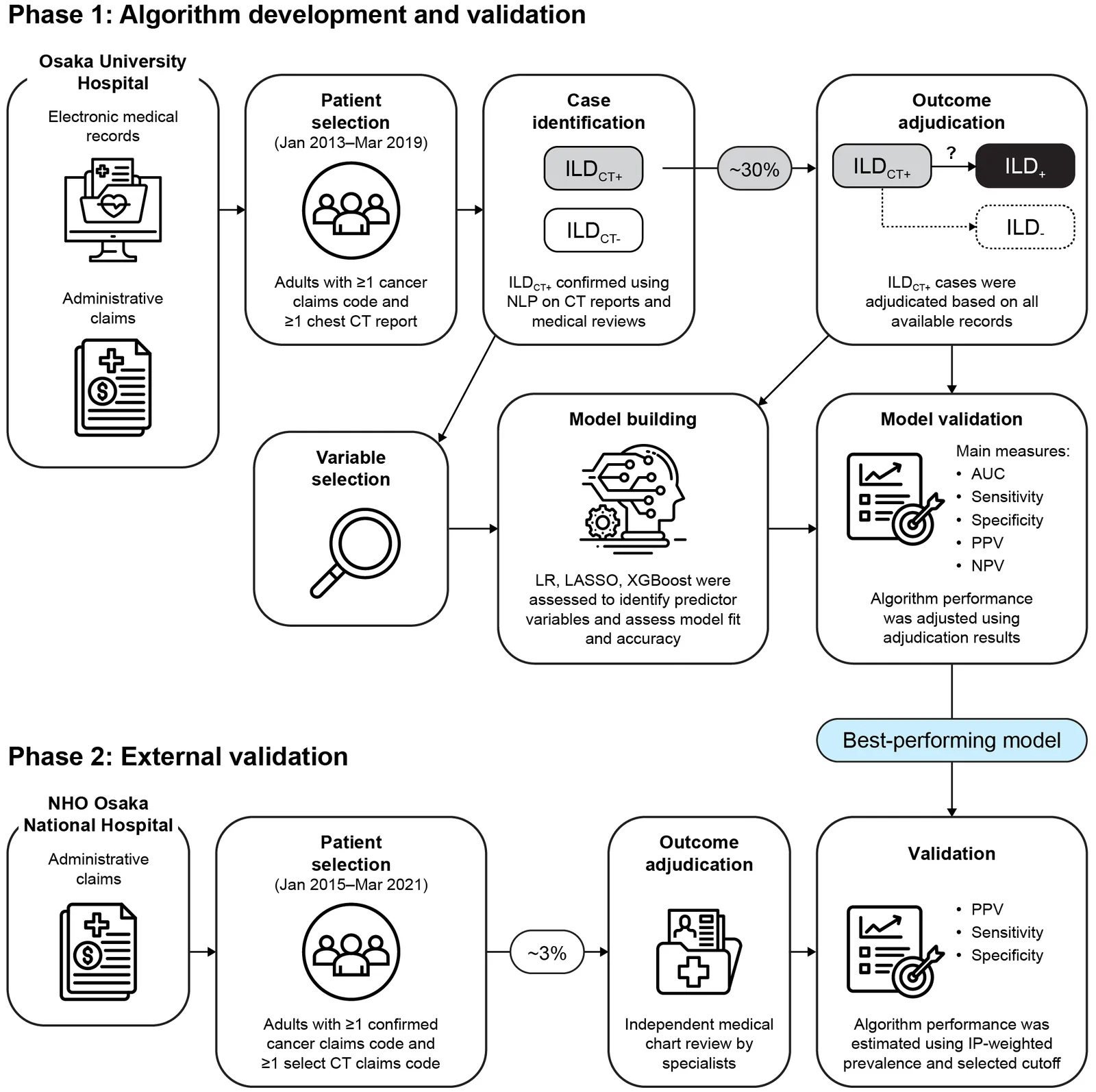
Study design. ILD_CT-_ cases were non-ILD cases with no ILD-related clinical findings based on NLP. ILD_+_ cases were confirmed ILD cases based on outcome adjudication. ILD- cases were non-ILD cases confirmed based on outcome adjudication. AUC, area under the curve; CT, computed tomography; ILD, interstitial lung disease; IP, inverse probability; LASSO, least absolute shrinkage and selection operator; LR, logistic regression; NLP, natural language processing; NPV, negative predictive value; PPV, positive predictive value; XGBoost, eXtreme Gradient Boosting.

This was an observational study that developed and validated claims-based algorithms using machine-learning modeling to identify ILD in Japanese patients with cancer in routine clinical practice (**Figure 1**). The study was conducted in two phases; the first phase (Phase 1) developed and validated the algorithm, and the second phase (Phase 2) assessed the algorithm’s generalizability and robustness in an external cohort. For Phase 1, patients with any cancer were selected using administrative claims and electronic medical record data from Osaka University Hospital. Osaka University Hospital is a tertiary care center in Japan that serves a diverse patient population from local communities and referral cases across Western Japan. Osaka University Hospital had approximately 1100 beds and 730 doctors in 2024; in 2023, the institution had a total of 564,217 outpatients and 320,574 inpatients. For Phase 2, the best-performing algorithm from Phase 1 was evaluated in patients who were selected using a claims database from NHO Osaka National Hospital. NHO Osaka National Hospital is a specialized tertiary center in Japan, as well as a cancer treatment hub serving as a referral center for complex cancer cases, offering advanced surgical interventions, radiation therapy, and specialized oncology services across multiple departments. The institution has a capacity of 605 beds. In 2023, the institution had 263 doctors; in 2022, there were 240,023 outpatients and 167,865 inpatients in total. This study was conducted in accordance with the Declaration of Helsinki and applicable Japanese laws and regulations. Study protocols were approved by the Non-Profit Organization MINS Research Ethics Committee on January 13, 2021 (MINS-REC-210201; Phase 1) and October 5, 2022 (MINS-REC-220228; Phase 2). Informed consent was not required because de-identified data were used.

### 2.2 Study population

For Phase 1, patients selected from claims data and electronic medical records were aged ≥18 years with at least two claims for any code types (e.g., disease, procedure, and medication codes) that were ≥30 days apart between January 1, 2013, and March 31, 2019; at least one claim for any cancer codes (excluding disease codes with a suspected flag, indicated by a specific code designation or the phrase “suspected diagnosis”) between January 1, 2013, and March 31, 2019; and at least one chest computed tomography (CT) report available in the electronic medical record between January 1, 2013, and March 31, 2019.

For Phase 2, patients selected from claims data were aged ≥18 years with at least one confirmed claim for any cancer code (i.e., cancer codes without any suspected flags) between January 1, 2015, and March 31, 2021, and at least one CT claims code available from the date after the initial cancer diagnosis to March 31, 2022. The CT claims codes included the Japanese standardized procedure codes 170011710, 170011810, 170028610, or 170033410, selected using all codes with the description of “CT imaging”, but excluding codes that could be used for purposes other than CT and those indicating that patients were referred to another medical facility for imaging.

### 2.3 Phase 1: claims-based algorithm development and validation

#### 2.3.1 Case identification

Patients were assigned to ILD cohorts or non-ILD controls based on diagnosis notes embedded in chest CT reports using natural language processing (NLP), which converts unstructured clinical text to structured/encoded data. The NLP system was developed by the Department of Medical Informatics at Osaka University and has been previously published (15, 16). Briefly, the NLP system used a comprehensive, predefined dictionary of ILD-related Japanese medical terms (e.g., interstitial pneumonia patterns, ground-glass opacities, reticular abnormalities, honeycombing, traction bronchiectasis, and related findings), which were selected and reviewed by clinical experts including a radiologist (**Supplementary Methods**). CT reports with no ILD-related clinical findings were assigned to the non-ILD (ILD_CT-_) cohort. NLP served as a screening tool for patient assignment, and therefore all potential ILD cases identified through NLP underwent a confirmatory medical review by three physicians, including a radiologist. ILD cases confirmed by medical review were denoted as ILD_CT+_; cases classified as suspected or non-ILD, or those not reaching physician consensus during the additional review, were excluded from further analyses. For the ILD_CT+_ cohort, the index date was defined as the earliest confirmed ILD diagnosis date recorded in the CT report. For the ILD_CT-_ cohort, one of the available CT report dates was randomly selected as the index date.

#### 2.3.2 Variable selection and model building

Candidate variables were extracted from claims data and included baseline demographics and diagnosis/disease codes (from any time before to 6 months after the index date) and procedure/medication codes (from 3 months before to 6 months after the index date). The extraction periods included 6 months after the index date, incorporating pre- and peri-diagnostic information into the model, to account for documentation and coding delays associated with claims data. Variables were selected based on two-step selection strategies. Firstly, variables were preselected based on their clinical relevance (3, 4), as determined by the medical experts. When direct data on specific risk factors were not available, proxy variables were used where possible. For example, baseline comorbidity data were used as a proxy measure for performance status data as they were not available from the database. Selected candidate variables included age, sex, prior ILD diagnosis codes, Charlson comorbidities, presence of pulmonary diseases (e.g., chronic obstructive pulmonary disease/emphysema, pleural effusion/pulmonary edema, pulmonary nontuberculous mycobacterial disease, and tuberculosis), and presence of any cancer by type (e.g., lung cancer/lung metastases), as well as procedure codes related to ILD diagnostics (e.g., Krebs von den Lungen-6 [KL-6] and serum surfactant protein-D [SP-D]), and medications previously associated with ILD (e.g., cancer treatments). Additional variables were identified based on statistical significance (p<0.05 using Fisher’s exact test or Wilcoxon signed-rank test for comparing ILD_CT+_ and ILD_CT-_ cohorts) and minimal frequency threshold (≥2% in both the ILD_CT+_ and ILD_CT-_ cohorts). All relevant codes are listed in the Phase 1 code list.

For model building, candidate variables were explored using three machine-learning modeling strategies (logistic regression [LR], least absolute shrinkage and selection operator [LASSO] logistic regression, and eXtreme Gradient Boosting [XGBoost]). These models were fitted and assessed for accuracy using 1000 random splits. In each split, patients were randomly allocated to the training sample (80%) for model fitting and the validation sample (20%) to evaluate ILD probability. The regularization hyperparameter lambda (for LASSO) and hyperparameters (for XGBoosts) were tuned within the training set using five-fold cross-validation to maximize the area under the curve (AUC). Specifically, a grid search was conducted to optimize the following XGBoost hyperparameters, which were selected based on prior domain knowledge and empirical testing to optimize the model’s performance: tree depth (max_depth: 3, 4, 5, 6, 7), learning rate (eta: 0.1, 0.01, 0.001), minimum child weight (min_child_weight: 1, 10, 100), subsample ratio (subsample: 0.5, 0.75, 1), and feature sampling ratio (colsample_bytree: 0.5, 0.75, 1).

Model performance measures (AUC, accuracy, sensitivity, specificity, positive predictive value [PPV], negative predictive value [NPV], Youden’s index, F-measure, and Matthew’s correlation coefficient) and variable importance measures were calculated on the validation set for each model where appropriate. Main analysis was conducted using pre-adjudicated outcomes (ILD_CT+_ and ILD_CT-_), a fixed cutoff value of 0.5 (representing a natural probability boundary), and an estimated ILD prevalence of 6.4% (based on preliminary data on an estimated prevalence of cyclin-dependent kinase 4/6 inhibitor-related ILD from a Japanese nested case-control study (17)). The cutoff was a threshold used to classify each observation based on its modeled probability; observations with a modeled probability greater than the cutoff were classified as positive cases, and those with a modeled probability less than the cutoff were classified as negative cases. For example, for the fixed cutoff value of 0.5, cases with >50% estimated probability of ILD were classified as positive. Sensitivity analyses were conducted using the pre-adjudicated and adjudicated outcomes, with other combinations of the following settings: fixed (0.5) or optimal (i.e., maximizing F-measure) cutoff values, and ILD prevalence of 6.4% or 10%. The reduced model, using only the top variables identified in the full model, was evaluated for its performance using the same settings and combinations used for the full model. Because the data extraction period for the variable selection included 6 months after the index date, to further assess the robustness of the chosen model, an additional sensitivity analysis was conducted by calculating model performance measures using only pre-index data for variable selection. No imputation was conducted as there were no missing values.

#### 2.3.3 Outcome adjudication and validation

Patients were randomly selected for outcome adjudication, conducted by two independent pulmonologists, based on available medical records. Adjudicated ILD cases were denoted as ILD_+_; cases with a different diagnosis between the pulmonologists involved a third specialist for discussion. Patients were excluded if no consensus was reached. Model performance measures were adjusted using the adjudication results (**Supplementary Figure 1**).

### 2.4 Phase 2: external validation

#### 2.4.1 Estimation of ILD probability

The probability of having ILD was estimated for each patient based on the “best-performing” claims-based algorithm from Phase 1 using a regression model. Because claims codes do not specify the exact anatomical site of a CT scan, the index date for Phase 2 was defined as the date of a CT-related claims code occurring after the initial cancer diagnosis and before data cutoff. If patients had multiple index dates, the date with the highest estimated ILD probability was selected as the targeted index date. Patients were classified as non-ILD cases if no chest CT report was documented in their electronic medical records (used for outcome adjudication) within ±6 months of the targeted index date. This classification approach was based on the understanding that a chest CT scan is essential for diagnosing ILD, and that a confirmed diagnosis is considered highly unlikely without a documented chest CT report. For patients with more than one chest CT report within ±6 months, the chest CT report nearest the targeted index date was used to confirm ILD status.

#### 2.4.2 Variables

Patient demographic and clinical information was collected from administrative claims. Confirmed ILD diagnosis was determined based on manual medical review of all available chart records, including chest CT reports around each CT claims code, other imaging reports, lung biopsy records, physicians’ notes, administration and discharge summary, and laboratory reports. All relevant codes are listed in the Phase 2 code list.

#### 2.4.3 Outcome adjudication and estimation of diagnostic accuracy measures

Outcome adjudication was conducted by independent specialists. To balance resource burden and efficiency, 189 (3.4%) of 5625 eligible patients were sampled using unequal probability sampling based on the estimated probability of having ILD as derived from the identification algorithm (described above), with the probabilities rescaled to reach the target sample size. For patients selected for adjudication, sampling weights were computed using inverse probability (IP) weighting to adjust for unequal selection probabilities. As the prevalence of ILD was unknown, a prespecified cutoff value could not be determined to classify adjudicated patients into the “algorithm=Yes” cohort (modeled probability greater than the cutoff) or the “algorithm=No” cohort (modeled probability equal to or lower than the cutoff). Sampling weights were used to estimate the IP-weighted ILD prevalence and to select a cutoff that ensured the IP-weighted proportion of patients classified as ILD cases approximated the IP-weighted prevalence. Diagnostic accuracy measures were estimated using the IP-weighted prevalence and the selected cutoff.

## 3 Results

### 3.1 Phase 1: claims-based algorithm development and validation

#### 3.1.1 Study population and baseline characteristics

**Figure 2:**
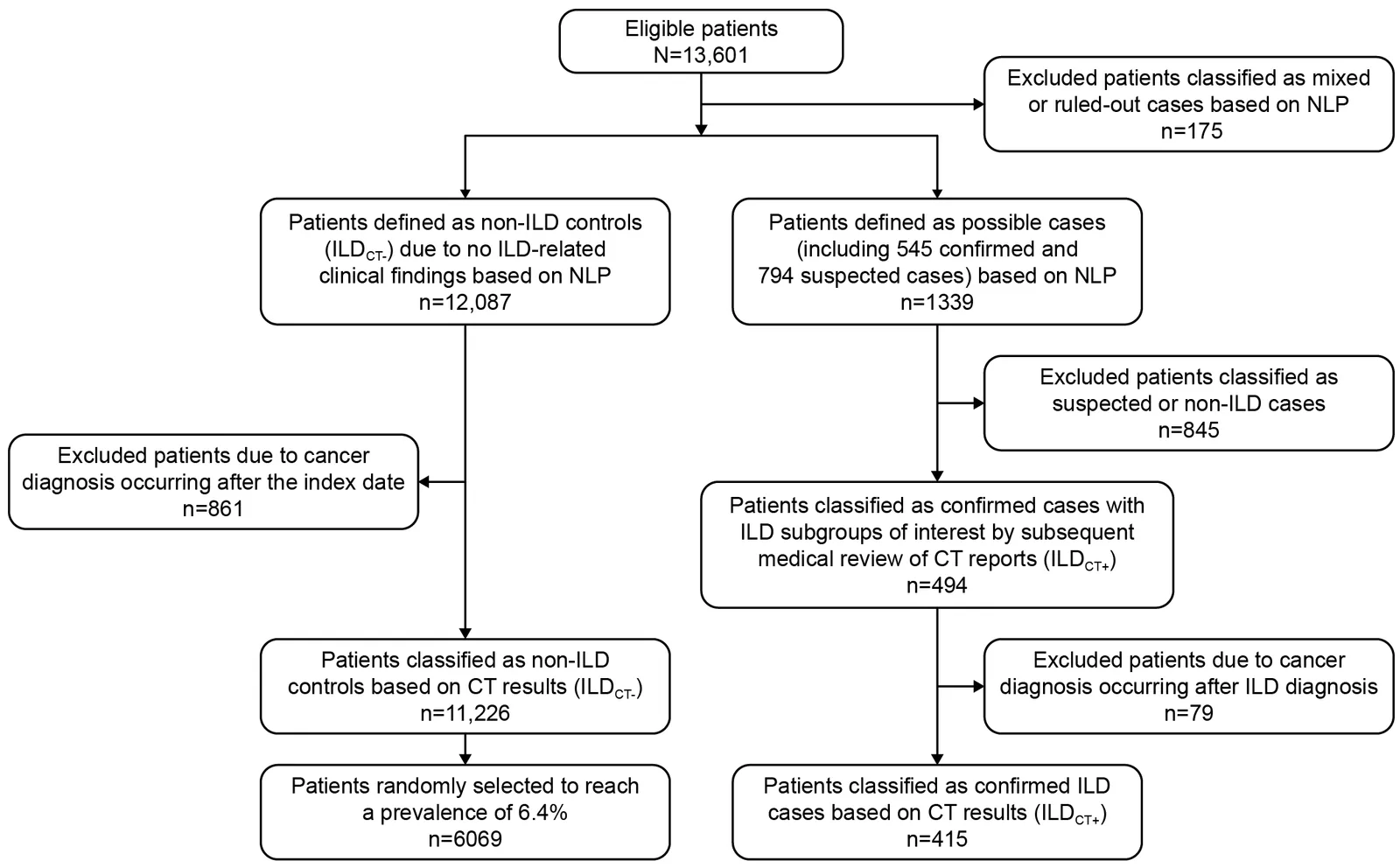
Flowchart of eligible patients in Phase 1. CT, computed tomography; ILD, interstitial lung disease; NLP, natural language processing.

Of 13,601 eligible patients, 415 were included in the ILD_CT+_ cohort (**Figure 2**). Among the ILD_CT-_ cohort, 6069 patients were randomly selected to reach the target ILD prevalence of 6.4%. Overall, the proportion of male and female patients was similar (**Table 1**). However, the ILD_CT+_ cohort was older, with a greater proportion of males, patients with lung cancer, and patients with comorbidities, than the ILD_CT-_ cohort.

**Table 1:** Baseline characteristics of eligible patients in Phase 1.

Characteristic	All patients (N=11,641)	ILD (ILD_CT+_) cohort^a^ (N=415)	Non-ILD (ILD_CT-_) cohort^b^ (N=11,226)
Sex			
Female	5516 (47.4)	120 (28.9)	5396 (48.1)
Male	6125 (52.6)	295 (71.1)	5830 (51.9)
Age, years			
Mean (SD)	65.4 (13.3)	72.6 (9.5)	65.2 (13.4)
Min, max	19, 104	33, 94	19, 104
Age group, years			
<60	3226 (27.7)	36 (8.7)	3190 (28.4)
60 to <70	3318 (28.5)	104 (25.1)	3214 (28.6)
70 to <80	3653 (31.4)	175 (42.2)	3478 (31.0)
≥80	1444 (12.4)	100 (24.1)	1344 (12.0)
Cancer type			
Lung cancer (including lung metastases)	2673 (23.0)	133 (32.0)	2540 (22.6)
Secondary malignant neoplasm of respiratory and digestive organs	1836 (15.8)	46 (11.1)	1790 (15.9)
Esophagus	1365 (11.7)	40 (9.6)	1325 (11.8)
Breast	1222 (10.5)	33 (8.0)	1189 (10.6)
Stomach	1130 (9.7)	49 (11.8)	1081 (9.6)
Charlson comorbidities^c^			
Peptic ulcer disease	3311 (28.4)	162 (39.0)	3149 (28.1)
Diabetes without chronic complications	2417 (20.8)	149 (35.9)	2268 (20.2)
Mild liver disease	1869 (16.1)	89 (21.4)	1780 (15.9)
Chronic pulmonary disease (including emphysema)	1797 (15.4)	151 (36.4)	1646 (14.7)
Congestive heart failure	1598 (13.7)	110 (26.5)	1488 (13.3)
Myocardial infarction	1493 (12.8)	91 (21.9)	1402 (12.5)
Cerebrovascular disease	1247 (10.7)	73 (17.6)	1174 (10.5)
Peripheral vascular disease	1038 (8.9)	70 (16.9)	968 (8.6)
Diabetes with chronic complications	801 (6.9)	58 (14.0)	743 (6.6)
Renal disease	637 (5.5)	42 (10.1)	595 (5.3)
Rheumatologic disease	499 (4.3)	68 (16.4)	431 (3.8)
Moderate or severe liver disease	206 (1.8)	9 (2.2)	197 (1.8)
Dementia	148 (1.3)	4 (1.0)	144 (1.3)
Hemiplegia or paraplegia	34 (0.3)	4 (1.0)	30 (0.3)
AIDS/HIV	13 (0.1)	1 (0.2)	12 (0.1)

AIDS, acquired immunodeficiency syndrome; CT, computed tomography; HIV, human immunodeficiency virus; ILD, interstitial lung disease; max, maximum; min, minimum; NLP, natural language processing; SD, standard deviation.

Data are reported as n (%) unless otherwise specified.

^a^ ILD cases as identified by NLP, supplemented with manual medical review of CT reports.

^b^ Non-ILD cohort, defined as patients with CT reports that did not contain any of the ILD-related clinical findings (i.e., a comprehensive list of predefined ILD-related medical terms).

^c^ Adapted from Quan H, et al., 2005 (23).

#### 3.1.2 Claims-based algorithm development

In the model assessment, 366 candidate variables (demographic: n=2; diagnosis-related: n=108; procedure-related: n=165; medication-related: n=91) were included.

**Figure 3:**
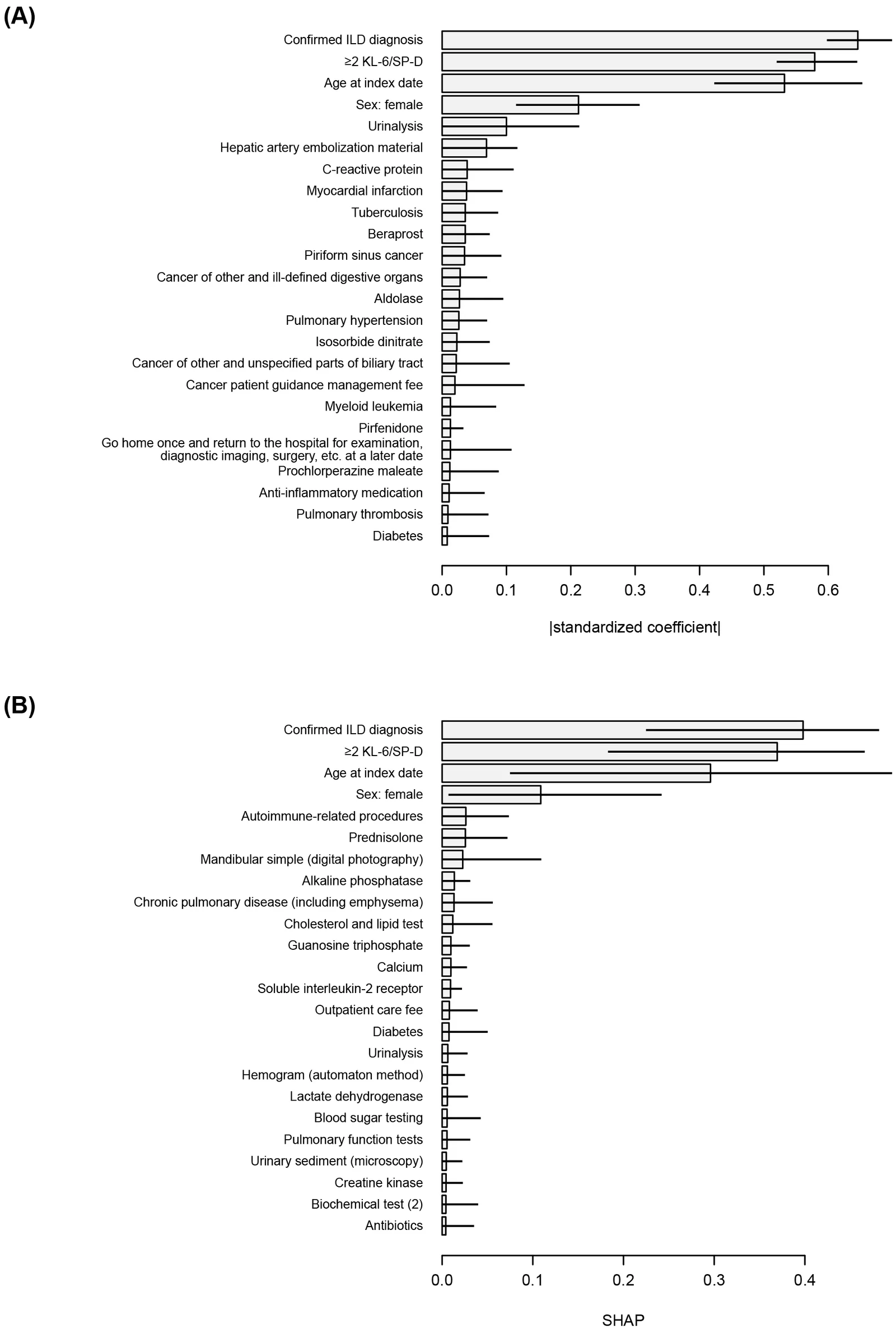
Variable importance plot for **(A)** LASSO, **(B)** XGBoost. These models were fitted and assessed for accuracy using 1000 random splits. Greater absolute standardized coefficient or SHAP values indicated greater variable importance (prediction for ILD). Error bars represent the 2.5th and 97.5th percentiles of the distribution of the absolute standardized coefficient or SHAP values. “Sex: female” showed a protective association, indicating that female sex was associated with lower ILD probability. ILD, interstitial lung disease; KL-6, Krebs von den Lungen-6; LASSO, least absolute shrinkage and selection operator; SHAP, SHapley Additive exPlanations; SP-D, surfactant protein-D; XGBoost, eXtreme Gradient Boosting.

In the main analysis, the top four variables identified using LASSO had notably higher variable importance than other variables, with the 2.5th and 97.5th percentiles not crossing zero, namely: confirmed ILD diagnosis codes; at least two KL-6/SP-D codes; age at index date; and female sex (protective association) (**Figure 3A**). Median AUC was 89.1%, median sensitivity was 39.8%, and median PPV was 75.6%, using pre-adjudicated outcomes, with a fixed cutoff value, and an ILD prevalence of 6.4% (**Table 2**). Similar performances were generally observed in the sensitivity analyses, which used the different combinations of settings (fixed [0.5] or optimal cutoff values, and ILD prevalence of 6.4% or 10%) (**Supplementary Table 1**). XGBoost showed similar performance and top four variables to LASSO (**Supplementary Tables 1**, **2**; **Figure 3B**). For LR, no analyses beyond the main analysis were performed because the modeled probabilities for at least one observation in the data were indistinguishable from 0 or 1.

**Table 2:** Main analysis: measures of model fit and accuracy for LASSO (full model)^a^.

Measure	Median	2.5th, 97.5th percentile	Min, max
Prevalence	6.4	6.4, 6.4	6.4, 6.4
Cutoff	0.5	0.5, 0.5	0.5, 0.5
AUC	89.1	85.2, 92.5	82.0, 94.5
True positive	33	25, 41	20, 47
False negative	50	42, 58	36, 63
False positive	11	5, 17	2, 23
True negative	1203	1197, 1209	1191, 1212
Sensitivity	39.8	30.1, 49.4	24.1, 56.6
Specificity	99.1	98.6, 99.6	98.1, 99.8
Accuracy	95.3	94.5, 96.1	94.1, 96.5
PPV	75.6	64.1, 87.5	58.2, 94.7
NPV	96.0	95.4, 96.6	95.0, 97.1
Youden	38.9	29.0, 48.3	23.0, 55.9
F-measure	52.0	41.7, 61.4	34.5, 67.6
MCC	52.6	43.0, 62.0	35.8, 67.3

AUC, area under the curve; LASSO, least absolute shrinkage and selection operator; max, maximum; MCC, Matthew’s correlation coefficient; min, minimum; NPV, negative predictive value; PPV, positive predictive value.

Data are reported as %, except for true positive, false negative, false positive, and true negative, which are reported as n.

^a^ Evaluated using 1000 random splits.

Given its ease of interpretation and reduced computational burden compared with XGBoost, the LASSO reduced model, which included only the top four variables identified from the full model, was selected as the best-performing model. The LASSO reduced model had a median AUC of 88.9–90.0%, median sensitivity of 39.1–60.9%, and median PPV of 57.1–78.8% across different settings (fixed [0.5] or optimal cutoff values, and ILD prevalence of 6.4% or 10%) (**Supplementary Table 3**). While some variabilities were observed, the performance of the LASSO reduced model was generally similar to the LASSO full model. Additionally, a sensitivity analysis that included only pre-index data for variable selection revealed model performance measures of fit and accuracy similar to the LASSO reduced model across different settings (median AUC: 84.7–85.9%; median sensitivity: 32.5–52.2%; median PPV: 50.0–71.4%).

#### 3.1.3 Claims-based algorithm validation

Among the ILD_CT+_ cohort, 123 (29.6%) cases were randomly selected for outcome adjudication. Of 37 cases identified as having ILD based on the final algorithm, 36 (97.3%) were confirmed as true ILD cases after adjudication (**Supplementary Figure 2A**). Of 86 cases classified as non-ILD cases based on the final algorithm, 81 (94.2%) were confirmed as true ILD cases after adjudication, indicating that these were ILD cases that were not captured by the algorithm (false negatives). Using the adjusted confusion matrix (**Supplementary Figure 1**), patient classification of pre-adjudicated outcomes was adjusted based on each patient’s estimated ILD probability and to reach the target prevalence (6.4%) (**Supplementary Figure 2B**). The final model had a sensitivity of 33.5%, specificity of 99.3%, PPV of 76.7%, and NPV of 95.8% after reweighing the validation cohort to the target prevalence of 6.4% and applying the adjudication-based correction factors (p1 = 0.97 for algorithm positive cases; p2 = 0.94 for algorithm-negative cases), as described in **Supplementary Figure 1**. The resulting adjudicated ILD count (ILD_+_) corresponds to a post-adjudication prevalence of 6.08% (394/6477), which reflects the adjudication correction applied to the 6.4%-reweighted cohort rather than a separate prevalence assumption (**Supplementary Figure 2C**). The final variables were confirmed ILD diagnosis codes, KL-6/SP-D codes, age, and sex.

### 3.2 Phase 2: external validation

#### 3.2.1 Study population and baseline characteristics

**Figure 4:**
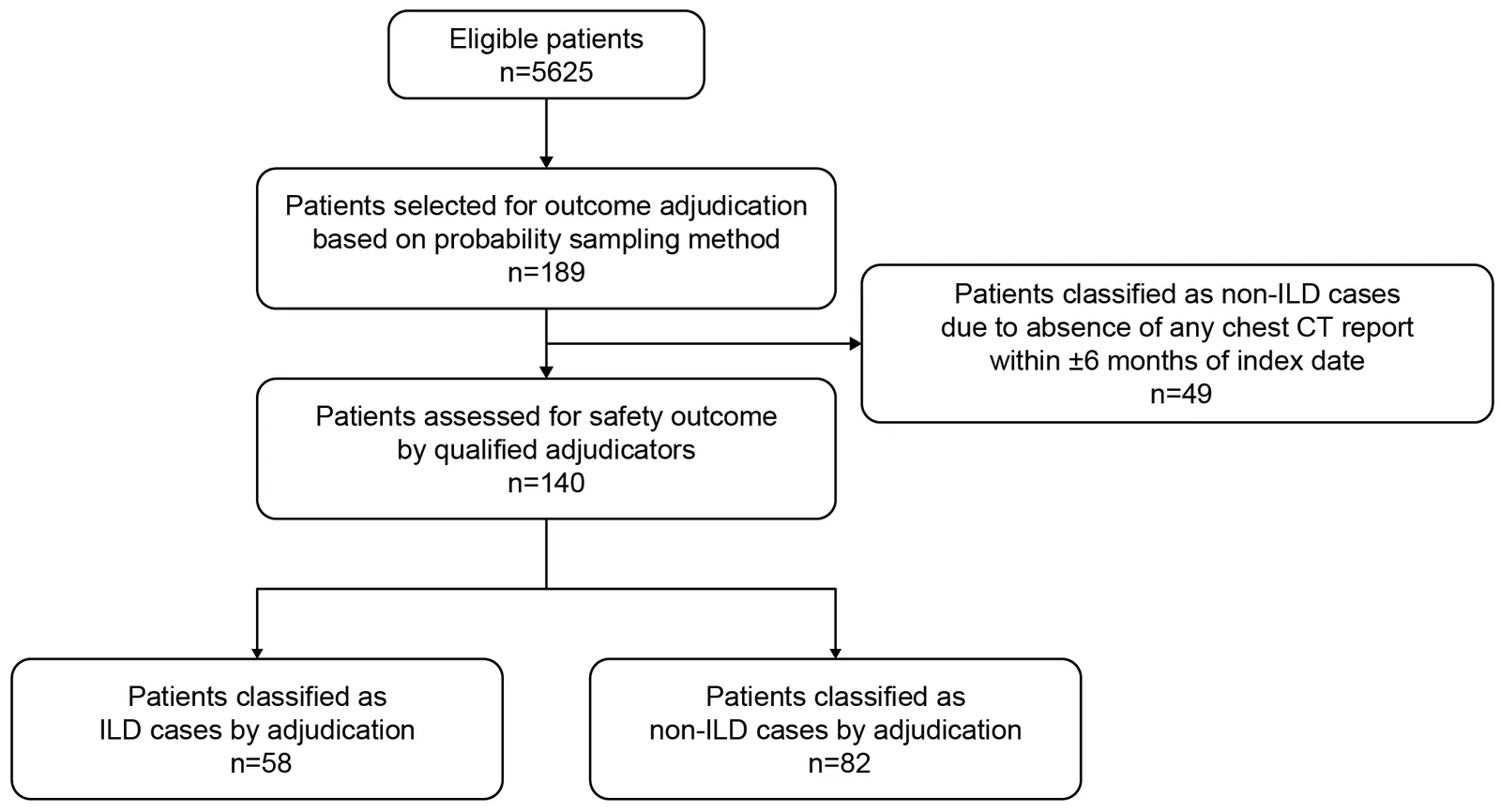
Flowchart of ILD classification following outcome adjudication in Phase 2. CT, computed tomography; ILD, interstitial lung disease.

Among 189 patients selected for outcome adjudication, 49 (25.9%) patients classified as non-ILD cases due to the absence of any chest CT report within ±6 months of the index date were excluded (**Figure 4**). Overall, 58 (41.4%) patients were classified as ILD cases by adjudication. Most patients (73.0%) were male, and mean age (standard deviation) was 75.2 (10.4) years (**Table 3**).

**Table 3:** Baseline characteristics of patients selected for outcome adjudication in Phase 2.

Characteristic	Patients selected for outcome adjudication (N=189)
Sex	
Female	51 (27.0)
Male	138 (73.0)
Age, years	
Mean (SD)	75.21 (10.42)
Min, max	39, 94
Age group, years	
<60	14 (7.4)
60 to <70	35 (18.5)
70 to <80	67 (35.4)
≥80	73 (38.6)

max, maximum; min, minimum; SD, standard deviation.

Data are reported as n (%) unless otherwise specified.

#### 3.2.2 Claims-based algorithm validation: ILD probability and outcome adjudication

The ILD prevalence in the adjudicated cohort was 30.7%. The IP-weighted prevalence of ILD was 5.9%. When a cutoff of 0.5 was used, 1.8% of patients had a modeled ILD probability that exceeded this cutoff; the claims-based algorithm had a sensitivity of 19.8%, specificity of 99.4%, and PPV of 65.5% (**Supplementary Table 4**).

## 4 Discussion

To our knowledge, this was the first observational study using machine-learning modeling to develop and validate a claims-based diagnostic algorithm that can identify ILD in Japanese patients with cancer. The best-performing algorithm was the LASSO reduced model, which, after adjustment based on adjudicated outcomes, had a PPV of 76.7%, specificity of 99.3%, and sensitivity of 33.5%. Identified variables were presence of confirmed ILD diagnosis codes, presence of KL-6/SP-D code, age, and sex. In an external database, this algorithm had a similar PPV (65.5%) and specificity (99.4%) but lower sensitivity (19.8%). The validated claims-based algorithm may enable confident identification of true positive cases of ILD when suspected positive from claims data in Japanese patients with cancer and be applicable as a case-confirmation tool for ILD that could be used in future retrospective studies, including PMS studies, that assess relative ILD risks between treatments. However, owing to its limited sensitivity, the algorithm may not reliably identify all true ILD cases and therefore would not be intended for a comprehensive ILD surveillance or absolute incidence estimation.

Ideally, algorithms used in PMS studies should have a moderate PPV balanced with sensitivity (18). Moreover, AUC, which measures the algorithm’s overall accuracy (19, 20), should be >0.80 for good accuracy and be considered clinically useful (21, 22). In this study, the final LASSO reduced model, chosen based on its high AUC, demonstrated an acceptable PPV (76.7%) but a relatively low sensitivity (33.5%). This limited sensitivity likely reflects the challenges in accurately identifying true ILD cases in claims data, where event misclassification, coding errors, and inconsistent recording of ILD diagnosis codes in the claims data may result in false negative cases in the model. When validated in another database (cutoff: 0.5), the model had a similar PPV (65.5%) but a lower sensitivity (19.8%). It is important to note that Phase 1 and Phase 2 served different purposes: Phase 1 developed and internally validated the algorithm, whereas Phase 2 assessed the performance of the validated algorithm under real-world conditions in an external cohort using a different case-identification approach. Therefore, the following differences, rather than the algorithm performance per se, may have contributed to the difference in sensitivity observed between the study phases. First, Phase 1 was performed using NLP, followed by a confirmatory review by three physicians, and excluded uncertain cases of ILD from outcome adjudication. Conversely, Phase 2 did not employ NLP for initial case screening. Phase 2 relied on the presence and absence of chest CT reports and included all potential ILD cases via manual chart review, including those in which ILD confirmation was difficult. Second, classification of ILD and non-ILD cases was different. In Phase 2, patients without a chest CT report within ±6 months of the targeted index date were classified as non-ILD, and because CT claims codes do not specify the anatomical site of the CT scan, some patients in Phase 2 may have been misclassified. Lastly, diagnosis and procedure codes used across the facilities varied, although this study was restricted to variables available in both facilities and could not fully assess the institutional variations. Therefore, further studies with careful model calibration and adaptation to data source characteristics are required to enhance case identification robustness. As PPV and NPV are prevalence dependent, PPV is expected to increase in populations with higher prevalence and decrease in populations with lower prevalence. In this study, the chosen ILD prevalence of 6.4% would have been a realistic estimate of the expected ILD in real-world clinical settings. In fact, although Phase 1 and 2 populations may have had a higher ILD prevalence than 6.4%, the sensitivity analysis using a higher prevalence of 10% also generally demonstrated consistent high PPV and further supported the robustness of the algorithm. Importantly, the algorithm’s relatively low sensitivity may underestimate ILD occurrence and therefore would not be suitable for estimating absolute ILD incidence rates as it may provide false reassurance about a drug’s safety. However, our intended application of this algorithm was to compare ILD risks between treatments. Given that PPV is prioritized in such an application to enhance cohort classification accuracy and to minimize false positives, the acceptable PPV of the final algorithm supports its potential real-world application as a case-confirmation tool in retrospective studies, particularly comparative pharmacoepidemiologic studies assessing ILD risks between certain drugs.

Our study based on machine-learning modeling objectively identified the top four variables (presence of confirmed ILD diagnosis codes, presence of KL-6/SP-D code, age, and sex) from 366 candidates. These variables were clinically intuitive and validate that the model captures meaningful clinical signals. The model provided probability estimates and therefore allows for adjustment of the cutoff threshold for different applications. Moreover, this study enabled rigorous quantification of the model’s performance through internal and external validation with outcome adjudication, differentiating itself from *ad hoc* rule-based algorithms.

To ensure comprehensive identification of key variables, this study employed a rigorous two-step approach for variable selection; variables were selected based on clinical relevance (e.g., risk factors for ILD (3, 4)), determined by medical experts. Subsequently, additional variables meeting statistical significance and frequency threshold were included, with the final set of variables included in the algorithm being ultimately selected based on the model’s empirical performance and accuracy across all candidate variables. Of the final set of variables, ILD diagnosis codes and KL-6/SP-D code may have originated from the same clinical pathway through which ILD cases were identified, and they suggest potential overlaps. However, given that ILD cases were determined by medical review of CT reports and confirmed by outcome adjudication using all available medical records, case identification and predictor variables would have captured different aspects of the clinical pathway and therefore would have not overestimated the specificity and PPV. As diagnoses at the index date often only appear in claims weeks or months later, this study included 6 months after the index date for the data extraction period to account for the data lag; this would have minimized key information being missed and improved the identification of patients with ILD, consistent with case finding and validation exercises, and represents the conditions under which the algorithm would be applied in retrospective database studies. It should be acknowledged that, in theory, the inclusion of the post-index period for data extraction may potentially cause data leakage and overfitting. However, the sensitivity analysis using pre-index data for variable selection in this study revealed similar model performance measures of fits and accuracy of the chosen model. The sensitivity analysis using pre-index data only had a median AUC of 84.7–85.9%, median sensitivity of 32.5–52.2%, and median PPV of 50.0–71.4% compared with the primary analysis that included both pre- and post-index data, which had a median AUC of 88.9–90.0%, median sensitivity of 39.1–60.9%, and median PPV of 57.1–78.8%. Therefore, the analysis that included pre-index data only retained meaningful predictive accuracy, indicating that the post-index data did not fundamentally alter the model performance and had minimal risk of data leakage, validating the robustness of the model. Nonetheless, because pre- and peri-diagnostic information is included, and because temporal ordering is relaxed, the validated model is not designed to function as a prospective prediction or a comprehensive surveillance tool nor to support real-time clinical decision-making; rather, as intended, the model serves as a case-confirmation tool for ILD in cancer patients, which may be particularly useful for real-world studies comparing the relative risk of ILD between cohorts using claims data.

Several factors may explain the discrepancies between the algorithm results and adjudication. Patients identified with ILD based on the claims-based algorithm but not on outcome adjudication may have had a considerable time interval between ILD diagnosis and adjudication, had ILD ruled out by testing (e.g., KL-6), or had an inaccurate diagnosis entered into medical records. Moreover, the absence of CT in the algorithm due to the study design (i.e., CT report data were used to confirm ILD diagnosis and index date in Phase 1), the lack of anatomical site specificity in the claims, and the differences in disease classification between the facilities may have also contributed to these discrepancies. Conversely, patients with confirmed ILD based on adjudication but not on the algorithm may have had no ILD diagnosis entry and no ILD confirmatory test recorded in the claims data.

This study used machine-learning modeling, which efficiently captured complex non-linear relationships between variables from two large databases, and developed and validated a claims-based algorithm for the identification of ILD in Japan. This study also assessed three machine-learning strategies using 1000 random splits and combinations of various settings, and it selected the best-performing algorithm to assess external validity. However, this study has several limitations. Owing to the nature of the claims, data may have lacked detailed clinical information (e.g., lung biopsy results), possibly limiting the precision and accuracy of identifying ILD. As CT claims do not specify the anatomical site of the CT reports, and as some patients may have had ILD diagnosed or managed at a referring institution, in Phase 2, patients without a documented chest CT report within ±6 months of the index date were classified as non-ILD cases, introducing outcome misclassification. However, this scenario is likely uncommon, as patients with cancer receiving active treatment at the study facility would generally be expected to undergo chest imaging at the same site as part of their routine oncologic care, particularly given that ILD is a recognized potential treatment-related adverse event that often prompts chest CT evaluation. Not all potential variables may have been available, key variables may have been inaccurate or misclassified, and coding variation was present due to differences in procedure code practices between facilities. Moreover, the definition of ILD used in this study does not distinguish drug-induced ILD from other pre-existing fibrotic changes (e.g., interstitial lung abnormalities). To enable a more detailed ILD subtype classification, future studies incorporating additional clinical data sources (e.g., pulmonary function test, pathology) may be required. The study may also be subject to selection bias as both phases were conducted at tertiary centers; generalizing the results to other, smaller hospitals and regions outside Japan requires caution and further validation. Calibration assessment was also out of scope for this study but could be considered in future applications of the algorithm. Importantly, the model was developed as a case-confirmation algorithm to identify ILD in patients with cancer and is not a prospective risk prediction tool.

In conclusion, the validated claims-based algorithm, with its acceptable PPV, may enable confident identification of true positive cases of ILD when suspected positive from claims data in Japanese patients with cancer. The algorithm may be valuable as a case-confirmation tool in future observational studies using healthcare databases, particularly in studies comparing the relative risk of ILD between cohorts. However, given its limited sensitivity, the algorithm may not be capable of capturing all true ILD cases and is therefore not intended for comprehensive ILD surveillance or absolute incidence estimation.

## Data Availability

The original contributions presented in the study are included in the article/**Supplementary Material**. Further inquiries can be directed to the corresponding author.
