# Supplementary material for: Development and validation of claims-based algorithms to identify interstitial lung disease among Japanese patients with cancer in routine clinical practice using real-world data sources
Source: Front Oncol. 2026 Sep 9;16:1750236. doi: 10.3389/fonc.2026.1750236 (PMC13597394; doi:10.3389/fonc.2026.1750236)
Supplement: Supplementary file 1 [file DataSheet1.docx]

SUPPLEMENTARY MATERIAL

LIST OF contents

supplementary methods

**SUPPLEMENTARY Table 1**	Measures of model fit and accuracy for LASSO (full model) and XGBoost.

**SUPPLEMENTARY Table 2**	Main analysis: measures of model fit and accuracy for XGBoost.

**SUPPLEMENTARY Table 3**	Measures of model fit and accuracy for LASSO (reduced model).

**SUPPLEMENTARY Table 4**	IP-weighted diagnostic accuracy measures.

**SUPPLEMENTARY Figure 1**	Adjusted metrics to measure the accuracy of adjudicated outcomes.

**SUPPLEMENTARY Figure 2	(A)** Confusion matrix based on 123 adjudicated cases, **(B)** Adjusted confusion matrix based on adjudicated outcomes (prevalence adjusted to 6.4%)

supplementary methods

Natural language processing (NLP) in Phase 1

The NLP system used in Phase 1 of this study for patient assignment was developed by the Department of Medical Informatics at Osaka University and has been previously published (1, 2). The NLP system used a comprehensive, predefined dictionary of ILD-related Japanese medical terms (e.g., interstitial pneumonia patterns, ground-glass opacities, reticular abnormalities, honeycombing, traction bronchiectasis, and related findings), which were selected and reviewed by clinical experts including a radiologist.

The overall system pipeline consisted of two sequential steps for extracting radiologic findings from free-text reports. First, the system converted each free-text radiology report into a predefined structured schema. Second, the structured outputs were matched with a predefined dictionary to identify ILD-related terms and phrases. The developed NLP system comprised entity extraction and certainty classification modules; in the entity extraction module, clinical terms, including observations such as “ground-glass opacities” and clinical findings such as “interstitial lung disease,” were extracted from free-text radiology reports. In the certainty classification module, each observation and clinical finding entity was assigned one of five diagnostic certainty levels: definite, likely, may represent, unlikely, or denial. A radiologic dictionary database, consisting of Japanese clinical terms and phrases related to ILD, was also constructed to identify ILD-related findings from the structured outputs generated by the NLP system.

In this study, candidate reports were defined using both diagnostic certainty and dictionary-based term matching. Among the terms and phrases extracted by the entity extraction module, candidate ILD-related reports were selected if the corresponding entity was assigned a certainty level other than “denial” and matched with one of the predefined ILD-related terms or phrases in the radiologic dictionary. These candidate reports were then reviewed by physicians for further evaluation.

References

1. Sugimoto K, Takeda T, Oh J-H, et al. Extracting clinical terms from radiology reports with deep learning. J Biomed Inform. (2021) 116:103729. doi: 10.1016/j.jbi.2021.103729.
2. Sugimoto K, Wada S, Konishi S, et al. Classification of diagnostic certainty in radiology reports with deep learning. Stud Health Technol Inform. (2024) 310:569–573. doi: 10.3233/shti231029.

SUPPLEMENTARY **Table 1**	Measures of model fit and accuracy for LASSO (full model) and XGBoost.^a^

| Model type | Cutoff^b^ | AUC | Sensitivity | Specificity | Accuracy | PPV | NPV | Youden | F-measure | MCC |
| --- | --- | --- | --- | --- | --- | --- | --- | --- | --- | --- |
| Pre-adjudicated (lLD_CT+_ or ILD_CT-_), set prevalence 6.4% | | | | | | | | | | |
| XGBoost | Fixed | 88.3  (84.8, 92.0) | 41.0  (28.9, 51.8) | 99.2  (98.6, 99.7) | 95.5  (94.6, 96.1) | 77.3  (65.6, 89.4) | 96.1  (95.3, 96.8) | 39.9  (28.4, 50.7) | 53.1  (42.0, 62.9) | 53.9  (43.8, 63.2) |
| LASSO | Fixed | 89.1  (85.2, 92.5) | 39.8  (30.1, 49.4) | 99.1  (98.6, 99.6) | 95.3  (94.5, 96.1) | 75.6  (64.1, 87.5) | 96.0  (95.4, 96.6) | 38.9  (29.0, 48.3) | 52.0  (41.7, 61.4) | 52.6  (43.0, 62.0) |
| XGBoost | Optimal | 88.3  (84.8, 92.0) | 56.6  (44.6, 67.5) | 97.8  (96.4, 98.8) | 95.1  (93.9, 96.1) | 63.5  (52.3, 75.3) | 97.1  (96.3, 97.8) | 54.4  (43.0, 64.9) | 59.6  (51.1, 67.1) | 57.4  (48.1, 65.2) |
| LASSO | Optimal | 89.1  (85.2, 92.5) | 54.2  (42.2, 66.3) | 97.8  (96.1, 98.8) | 94.9  (93.7, 95.9) | 62.3  (50.6, 74.7) | 96.9  (96.1, 97.7) | 51.5  (39.9, 63.1) | 57.3  (49.0, 65.3) | 54.9  (46.2, 63.6) |
| Adjudicated (lLD_+_ or ILD_CT-_), set prevalence 6.4% | | | | | | | | | | |
| XGBoost | Fixed | 88.5  (79.9, 95.5) | 34.8  (17.4, 52.2) | 99.1  (98.0, 100.0) | 95.1  (93.4, 96.4) | 73.3  (46.2, 100.0) | 95.8  (94.6, 96.9) | 33.9  (15.6, 51.6) | 46.7  (24.2, 64.7) | 48.2  (26.9, 64.8) |
| LASSO | Fixed | 89.0  (80.9, 95.4) | 39.1  (21.7, 52.2) | 99.1  (97.7, 99.7) | 95.1  (93.4, 96.4) | 71.4  (47.4, 92.9) | 96.0  (94.9, 96.9) | 37.1  (20.3, 51.9) | 48.6  (30.3, 64.9) | 49.8  (30.2, 65.3) |
| XGBoost | Optimal | 88.5  (79.9, 95.5) | 56.5  (30.4, 78.3) | 96.8  (93.0, 99.1) | 94.2  (91.2, 96.4) | 55.2  (36.8, 76.5) | 97.1  (95.5, 98.5) | 54.2  (30.1, 76.5) | 55.6  (38.7, 72.3) | 53.1  (35.0, 70.5) |
| LASSO | Optimal | 89.0  (80.9, 95.4) | 56.5  (34.8, 78.3) | 96.8  (93.0, 98.8) | 94.2  (91.0, 96.2) | 53.6  (36.4, 75.0) | 97.1  (95.7, 98.5) | 53.9  (31.9, 75.0) | 54.5  (38.1, 70.0) | 52.1  (34.5, 68.8) |
| Pre-adjudicated (lLD_CT+_ or ILD_CT-_), set prevalence 10% | | | | | | | | | | |
| XGBoost | Fixed | 88.4  (84.5, 92.0) | 48.2  (34.9, 60.2) | 98.7  (97.5, 99.5) | 93.5  (92.3, 94.8) | 79.6  (67.9, 90.9) | 94.4  (93.2, 95.7) | 46.3  (34.1, 58.4) | 59.5  (48.7, 69.5) | 58.6  (48.7, 68.1) |
| LASSO | Fixed | 89.1  (85.2, 92.5) | 44.6  (33.7, 54.2) | 98.8  (97.9, 99.5) | 93.3  (92.2, 94.5) | 80.4  (69.0, 91.2) | 94.1  (93.1, 95.1) | 43.0  (32.8, 53.1) | 56.9  (46.9, 65.7) | 56.6  (47.3, 64.9) |
| XGBoost | Optimal | 88.4  (84.5, 92.0) | 60.2  (48.2, 71.1) | 97.1  (95.2, 98.5) | 93.3  (91.6, 94.7) | 69.4  (58.5, 80.8) | 95.6  (94.4, 96.8) | 57.0  (45.5, 67.5) | 64.0  (55.1, 71.8) | 60.6  (51.2, 69.2) |
| LASSO | Optimal | 89.1  (85.2, 92.5) | 59.0  (45.8, 69.9) | 96.7  (94.5, 98.3) | 92.9  (91.2, 94.5) | 66.2  (55.7, 77.6) | 95.5  (94.1, 96.6) | 55.7  (43.0, 65.7) | 62.3  (53.3, 70.1) | 58.5  (49.9, 67.6) |
| Adjudicated (lLD_+_ or ILD_CT-_), set prevalence 10% | | | | | | | | | | |
| XGBoost | Fixed | 88.6  (80.2, 95.1) | 39.1  (21.7, 65.2) | 98.6  (96.2, 100.0) | 93.2  (90.6, 95.3) | 76.9  (55.0, 100.0) | 93.8  (92.1, 96.3) | 39.1  (21.3, 63.3) | 54.1  (33.3, 72.0) | 53.6  (34.0, 70.7) |
| LASSO | Fixed | 89.1  (81.8, 95.1) | 39.1  (21.7, 60.9) | 98.6  (96.7, 100.0) | 92.7  (90.6, 94.9) | 76.9  (53.3, 100.0) | 93.7  (92.1, 95.8) | 38.2  (20.8, 59.0) | 51.6  (33.3, 68.6) | 52.2  (33.3, 68.2) |
| XGBoost | Optimal | 88.6  (80.2, 95.1) | 60.9  (39.1, 82.6) | 95.7  (90.5, 98.6) | 92.3  (88.5, 95.3) | 60.0  (42.9, 83.3) | 95.8  (93.6, 98.1) | 58.0  (36.8, 78.3) | 61.2  (45.5, 75.0) | 57.1  (40.9, 73.0) |
| LASSO | Optimal | 89.1  (81.8, 95.1) | 60.9  (39.1, 82.6) | 95.3  (91.0, 98.6) | 92.3  (88.5, 94.9) | 60.0  (43.3, 80.0) | 95.8  (93.6, 98.0) | 57.1  (35.4, 76.9) | 60.5  (45.0, 73.9) | 56.6  (40.1, 71.3) |

AUC, area under the curve; ILD, interstitial lung disease; LASSO, least absolute shrinkage and selection operator; MCC, Matthew’s correlation coefficient; NPV, negative predictive value; PPV, positive predictive value; XGBoost, eXtreme Gradient Boosting.

Data are reported as % (median [2.5th percentile, 97.5th percentile]).

^a^Evaluated using 1000 random splits.

^b^Fixed cutoff was 0.5; optimal cutoff was selected through cross-validation to maximize F-measures.

**SUPPLEMENTARY Table 2**	Main analysis: measures of model fit and accuracy for XGBoost.^a^

|  | Median | 2.5th, 97.5th percentile | Min, max |
| --- | --- | --- | --- |
| Prevalence | 6.4 | 6.4, 6.4 | 6.4, 6.4 |
| Cutoff | 0.5 | 0.5, 0.5 | 0.5, 0.5 |
| AUC | 88.3 | 84.8, 92.0 | 80.8, 94.3 |
| True positive | 34 | 24, 43 | 17, 47 |
| False negative | 49 | 40, 59 | 36, 66 |
| False positive | 10 | 4, 17 | 2, 23 |
| True negative | 1204 | 1197, 1210 | 1191, 1212 |
| Sensitivity | 41.0 | 28.9, 51.8 | 20.5, 56.6 |
| Specificity | 99.2 | 98.6, 99.7 | 98.1, 99.8 |
| Accuracy | 95.5 | 94.6, 96.1 | 94.2, 96.6 |
| PPV | 77.3 | 65.6, 89.4 | 60.5, 95.3 |
| NPV | 96.1 | 95.3, 96.8 | 94.8, 97.1 |
| Youden | 39.9 | 28.4, 50.7 | 20.2, 55.7 |
| F-measure | 53.1 | 42.0, 62.9 | 32.7, 67.2 |
| MCC | 53.9 | 43.8, 63.2 | 38.4, 67.6 |

AUC, area under the curve; max, maximum; MCC, Matthew’s correlation coefficient; min, minimum; NPV, negative predictive value; PPV, positive predictive value; XGBoost, eXtreme Gradient Boosting.

Data are reported as %, except for true positive, false negative, false positive, and true negative, which are reported as n.

^a^Evaluated using 1000 random splits.

**SUPPLEMENTARY Table 3**	Measures of model fit and accuracy for LASSO (reduced model).^a^

| Cutoff | AUC | Sensitivity | Specificity | Accuracy | PPV | NPV | Youden | F-measure | MCC |
| --- | --- | --- | --- | --- | --- | --- | --- | --- | --- |
| Pre-adjudicated (lLD_CT+_ or ILD_CT-_), set prevalence 6.4% | | | | | | | | | |
| Fixed | 88.9  (85.3, 92.4) | 39.8  (30.1, 49.4) | 99.0  (98.4, 99.5) | 95.2  (94.4, 95.9) | 73.2  (62.2, 85.7) | 96.0  (95.4, 96.6) | 38.9  (29.3, 48.9) | 51.7  (41.7, 60.6) | 51.9  (42.1, 60.5) |
| Optimal | 88.9  (85.3, 92.4) | 53.0  (41.0, 65.1) | 97.9  (96.0, 98.8) | 94.9  (93.5, 95.9) | 62.2  (49.5, 73.7) | 96.8  (96.1, 97.6) | 50.9  (39.3, 62.3) | 57.1  (47.9, 64.9) | 54.6  (45.3, 63.0) |
| Adjudicated (lLD_+_ or ILD_CT-_), set prevalence 6.4% | | | | | | | | | |
| Fixed | 90.0  (82.9, 95.7) | 39.1  (21.7, 56.5) | 99.1  (97.7, 100.0) | 95.1  (93.7, 96.4) | 71.8  (50.0, 100.0) | 96.0  (94.9, 97.1) | 37.4  (20.0, 54.8) | 48.6  (29.4, 65.0) | 49.8  (30.2, 66.0) |
| Optimal | 90.0  (82.9, 95.7) | 56.5  (39.1, 78.3) | 97.1  (94.4, 98.8) | 94.5  (92.3, 96.4) | 57.1  (41.2, 77.8) | 97.1  (95.9, 98.5) | 54.5  (35.9, 75.3) | 57.1  (42.6, 72.3) | 54.7  (38.6, 70.5) |
| Pre-adjudicated (lLD_CT+_ or ILD_CT-_), set prevalence 10% | | | | | | | | | |
| Fixed | 89.0  (85.2, 92.4) | 47.0  (37.3, 56.6) | 98.7  (97.6, 99.5) | 93.4  (92.2, 94.6) | 78.8  (67.3, 90.0) | 94.4  (93.4, 95.3) | 45.6  (36.0, 55.0) | 58.8  (49.6, 67.2) | 57.9  (48.3, 66.0) |
| Optimal | 89.0  (85.2, 92.4) | 59.0  (47.0, 69.9) | 96.5  (94.9, 98.1) | 92.8  (91.2, 94.2) | 65.5  (56.2, 76.6) | 95.5  (94.3, 96.6) | 56.1  (44.0, 66.0) | 62.1  (53.6, 69.6) | 58.5  (49.6, 66.5) |
| Adjudicated (lLD_+_ or ILD_CT-_), set prevalence 10% | | | | | | | | | |
| Fixed | 89.9  (83.0, 95.8) | 43.5  (26.1, 65.2) | 98.6  (96.2, 100.0) | 93.2  (91.0, 95.7) | 76.5  (56.2, 100.0) | 94.2  (92.5, 96.3) | 43.0  (25.1, 63.8) | 56.4  (37.5, 73.5) | 55.5  (37.8, 73.4) |
| Optimal | 89.9  (83.0, 95.8) | 60.9  (39.1, 82.6) | 96.2  (91.5, 98.6) | 92.7  (89.3, 95.3) | 63.6  (46.1, 83.3) | 95.8  (93.6, 98.1) | 57.6  (36.7, 78.4) | 61.9  (47.1, 75.6) | 58.3  (42.2, 73.2) |

AUC, area under the curve; ILD, interstitial lung disease; LASSO, least absolute shrinkage and selection operator; MCC, Matthew’s correlation coefficient; NPV, negative predictive value; PPV, positive predictive value.

Data are reported as % (median [2.5th percentile, 97.5th percentile]).

^a^Evaluated using 1000 random splits.

**SUPPLEMENTARY Table 4**	IP-weighted diagnostic accuracy measures.

| Cutoff | 0 | 0.1 | 0.2 | 0.3 | 0.4 | 0.5 | 0.6 | 0.7 | 0.8 | 0.9 |
| --- | --- | --- | --- | --- | --- | --- | --- | --- | --- | --- |
| Prevalence in the adjudicated cohort | 30.7 | | | | | | | | | |
| IP-weighted prevalence | 5.9 | 5.9 | 5.9 | 5.9 | 5.9 | 5.9 | 5.9 | 5.9 | 5.9 | 5.9 |
| IP-weighted proportion of patients with  a predictive probability of ILD greater than the cutoff | 100.0 | 6.0 | 4.0 | 2.2 | 1.8 | 1.8 | 1.4 | 1.1 | 0.5 | 0 |
| IP-weighted PPV | 5.9 | 32.4 | 43.3 | 59.9 | 63.7 | 65.5 | 72.6 | 70.7 | 74.0 | NA |
| IP-weighted sensitivity | 100.0 | 33.4 | 29.3 | 22.8 | 19.8 | 19.8 | 17.8 | 13.6 | 6.9 | 0 |
| IP-weighted specificity | 0 | 95.7 | 97.6 | 99.1 | 99.3 | 99.4 | 99.6 | 99.7 | 99.8 | 100.0 |

ILD, interstitial lung disease; IP, inverse probability; NA, not applicable; PPV, positive predictive value.

Data are reported as %.

**SUPPLEMENTARY Figure 1**	Adjusted metrics to measure the accuracy of adjudicated outcomes. ILD_+_ cases were confirmed ILD cases based on outcome adjudication. ILD_CT-_ cases were non-ILD cases with no ILD-related clinical findings based on NLP. ILD, interstitial lung disease; NLP, natural language processing; NPV, negative predictive value; PPV, positive predictive value.

**SUPPLEMENTARY Figure 2**	**(A)** Confusion matrix based on 123 adjudicated cases among the ILD_CT+_ cohort, **(B)** Unadjusted confusion matrix based on pre-adjudicated outcomes (prevalence adjusted to ~6.4%), **(C)** Adjusted confusion matrix based on adjudicated outcomes (prevalence adjusted to 6.4%). ^a^ILD cases as identified by NLP supplemented with manual review of CT reports. ^b^Non-ILD cohort (defined as patients with CT reports that did not contain any of the ILD-related clinical findings). ^c^The count shown here differs marginally from the controls in Figure 2 (n=6069), due to rounding in the prevalence-adjustment computation; both correspond to the target ILD prevalence of 6.4%, and the difference does not affect the reported diagnostic accuracy measures. ^d^ILD cases based on adjudication results. CT, computed tomography; ILD, interstitial lung disease.
